# Supplementary figures and images for: Functional duality in group criticality via ambiguous interactions
Source: PLoS Comput Biol. 2023 Feb 15;19(2):e1010869. doi: 10.1371/journal.pcbi.1010869 (PMC9931117; doi:10.1371/journal.pcbi.1010869)

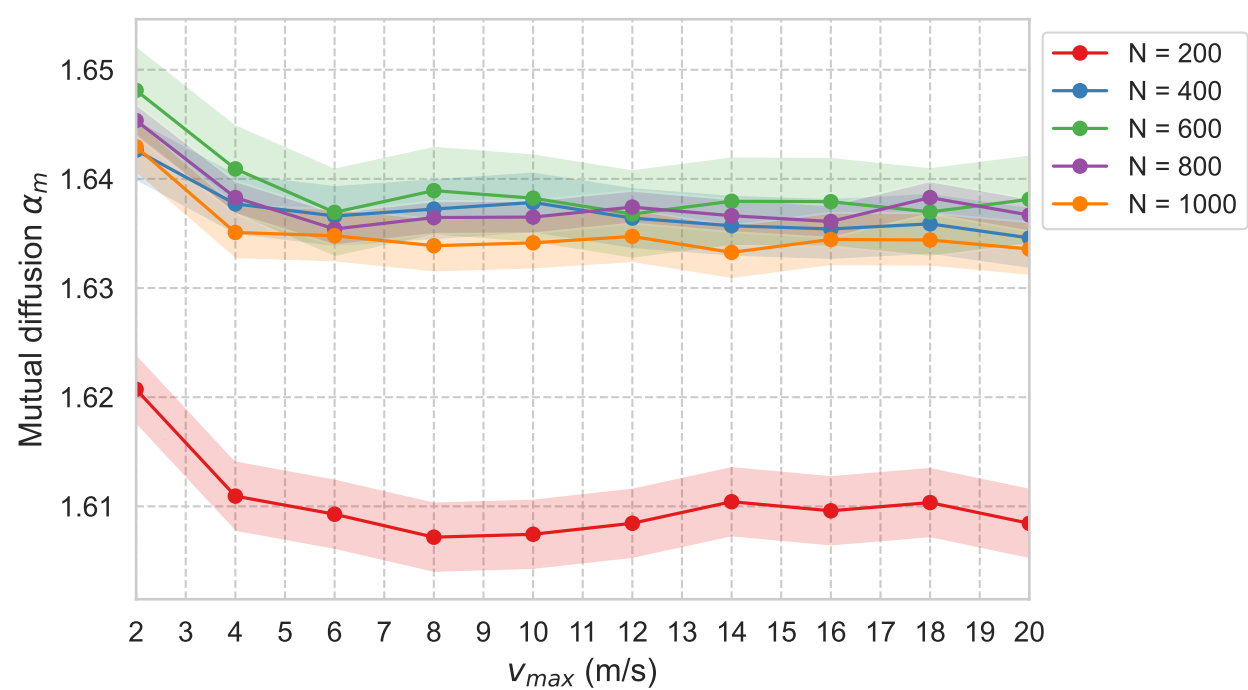

Supplement: S1 Fig — (PDF) [file pcbi.1010869.s001.pdf]

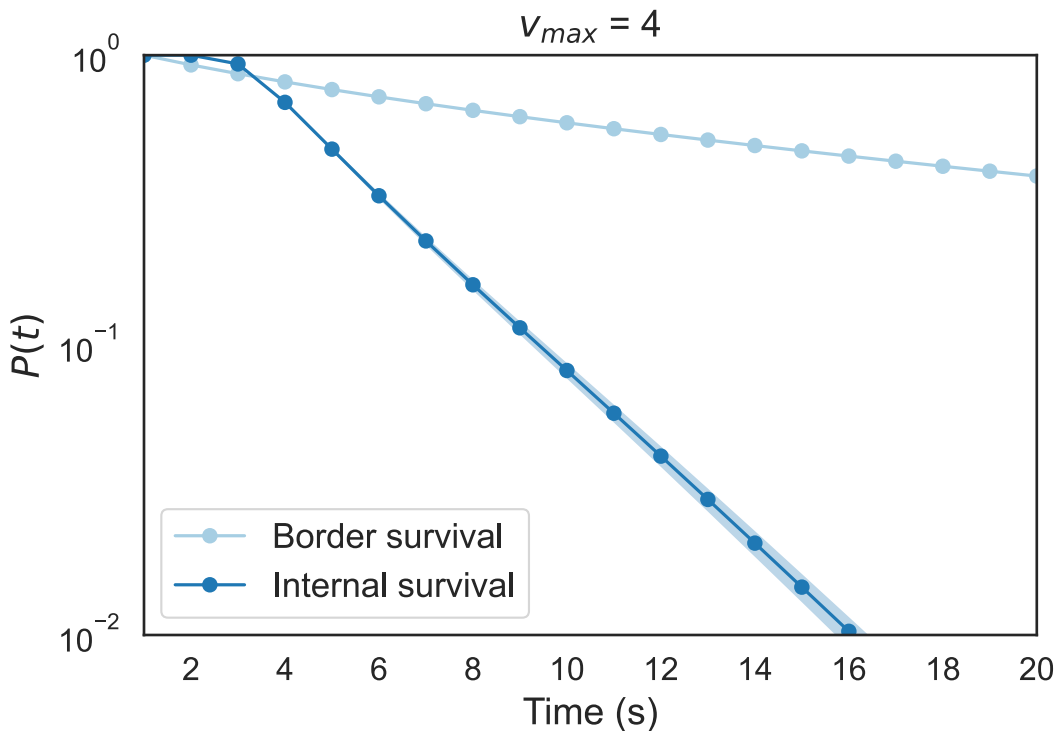

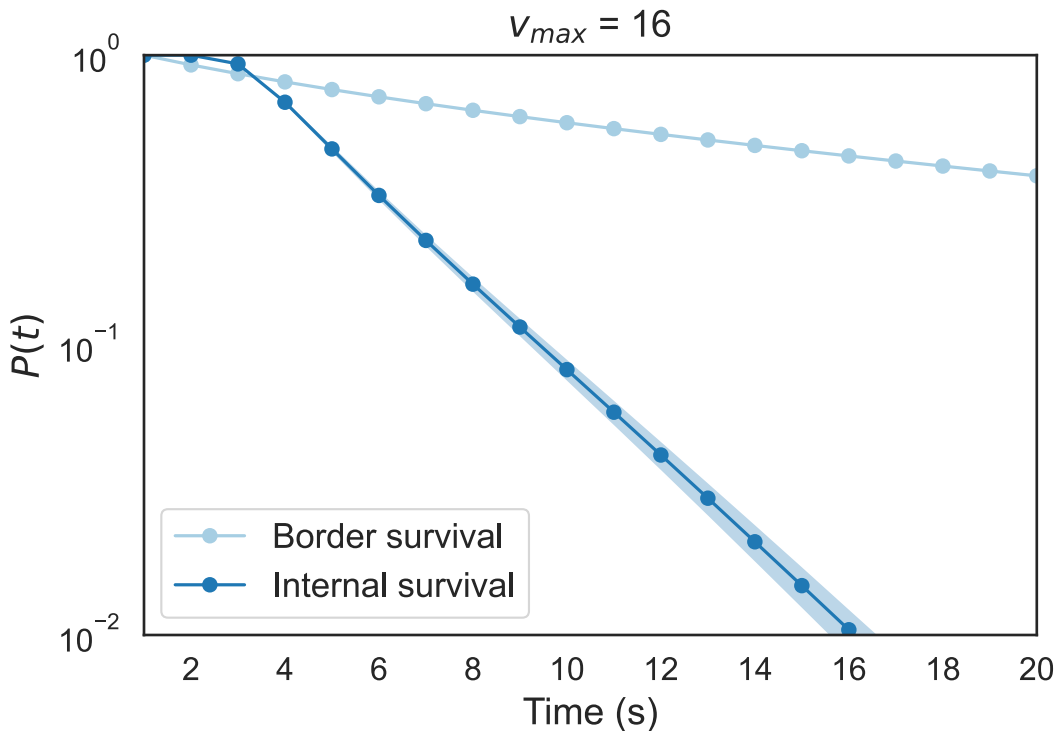

Supplement: S2 Fig — (PDF) [file pcbi.1010869.s002.pdf]

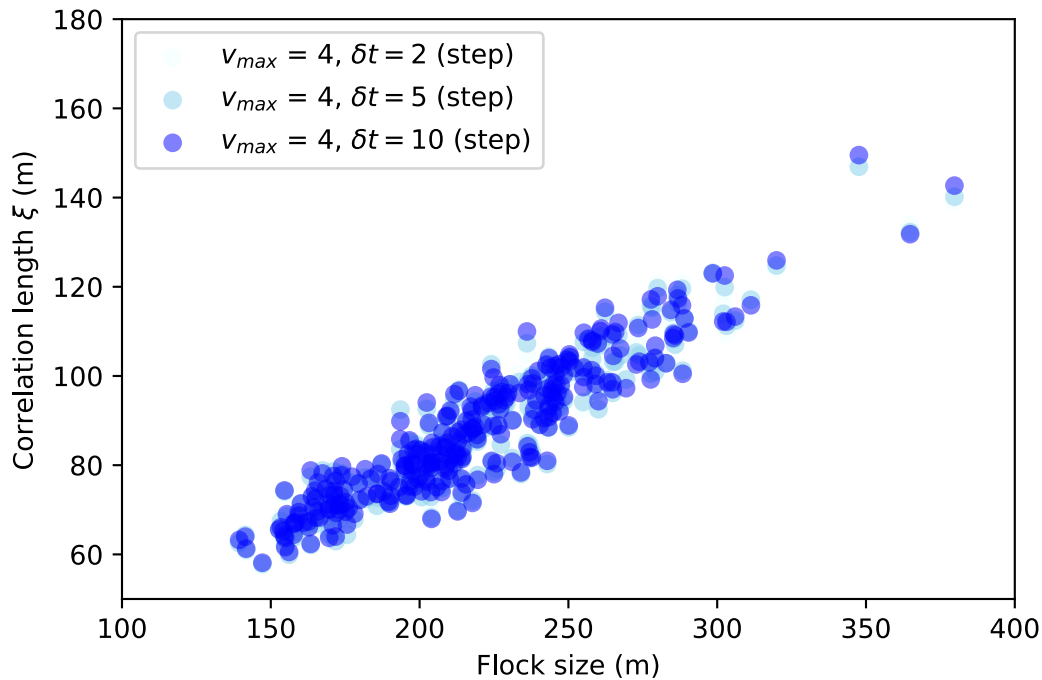

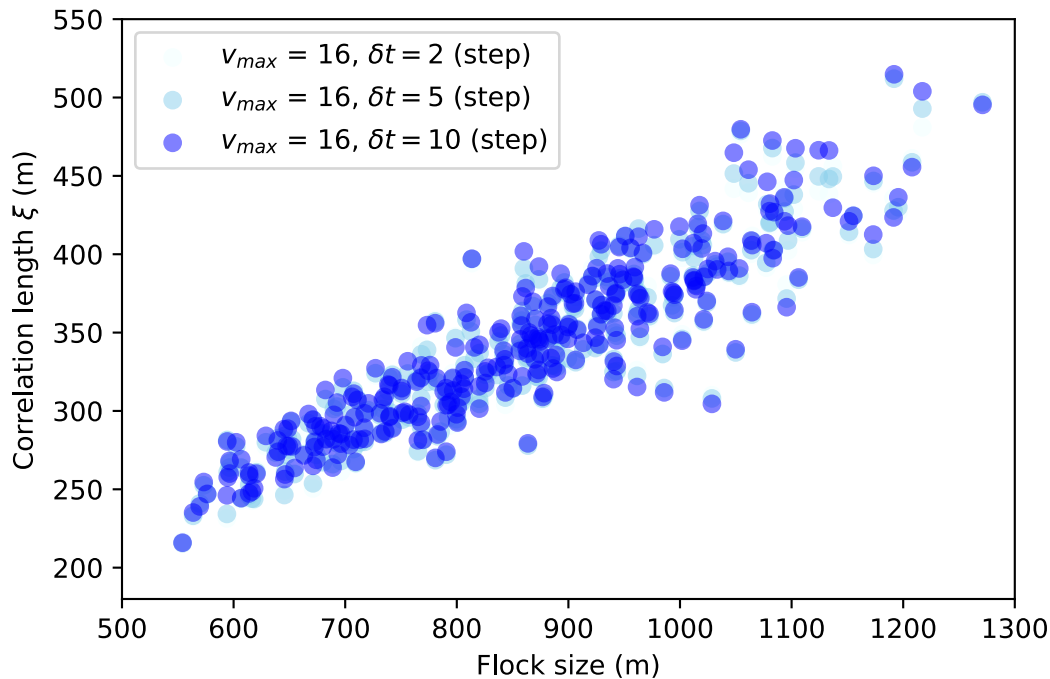

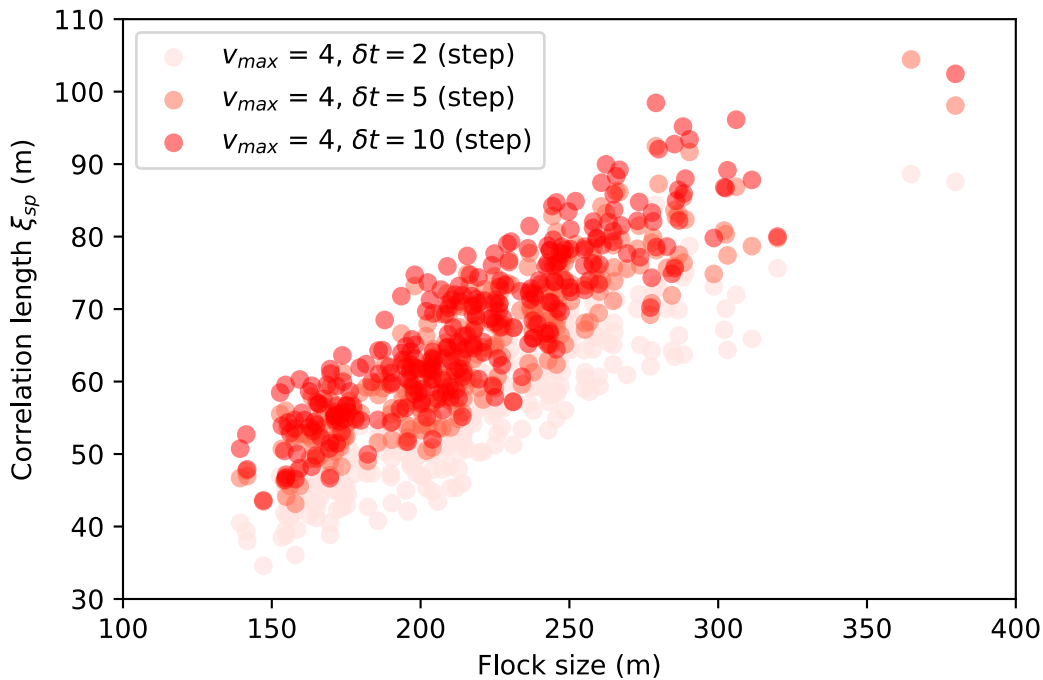

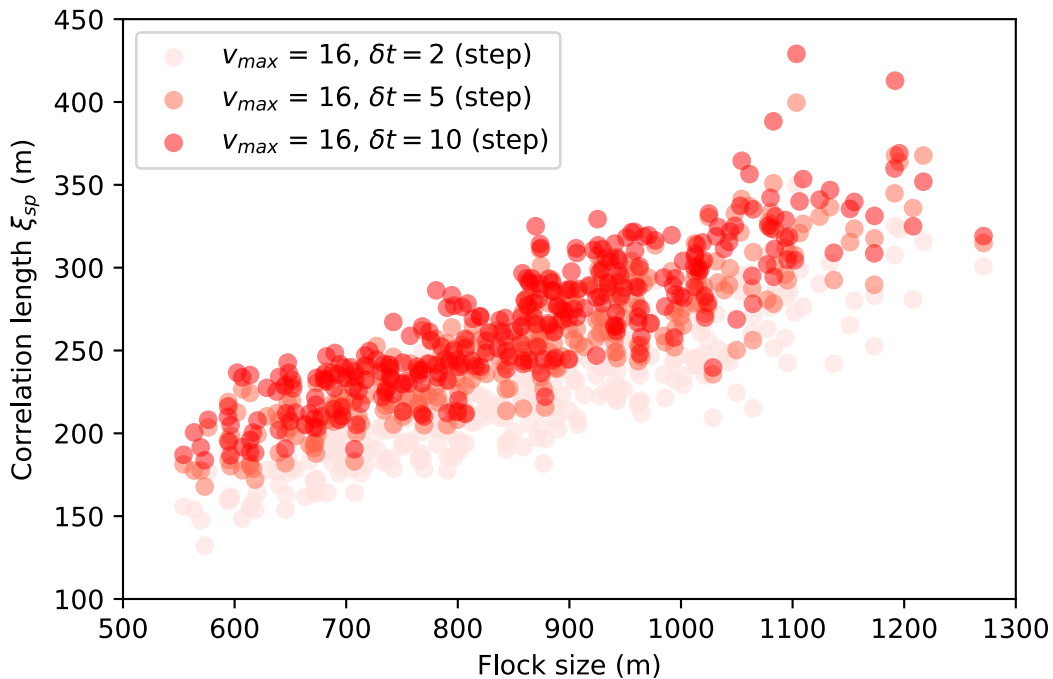

Supplement: S3 Fig — (PDF) [file pcbi.1010869.s003.pdf]

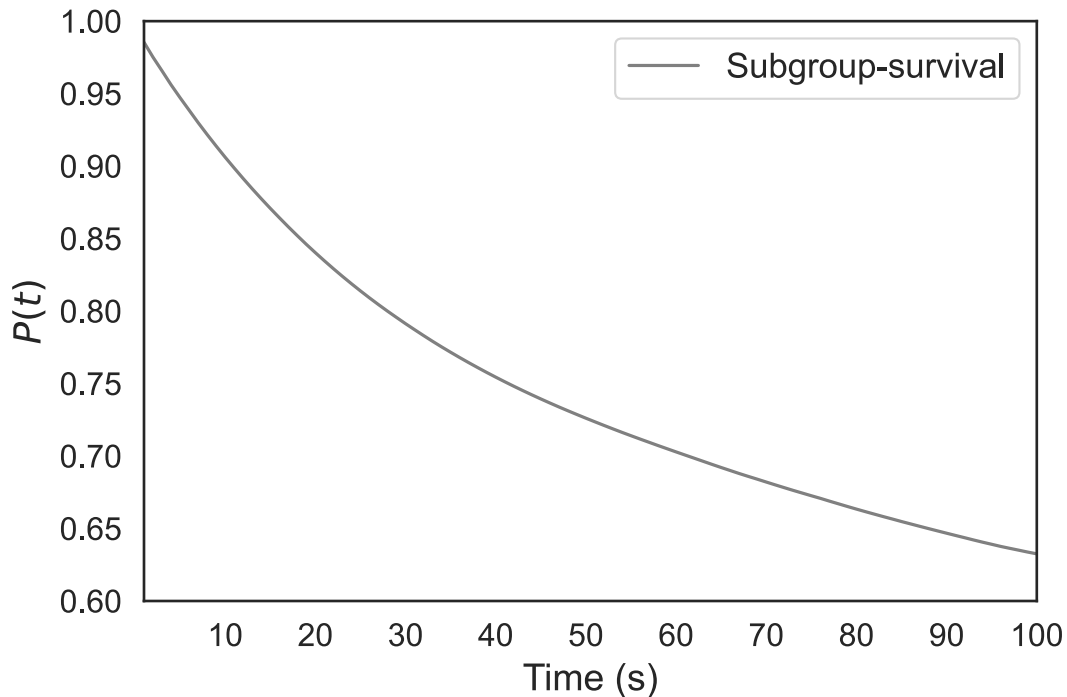

Supplement: S5 Fig — (PDF) [file pcbi.1010869.s005.pdf]
