# Supplementary material for: Functional duality in group criticality via ambiguous interactions
Source: PLoS Comput Biol. 2023 Feb 15;19(2):e1010869. doi: 10.1371/journal.pcbi.1010869 (PMC9931117; doi:10.1371/journal.pcbi.1010869)
Supplement: S4 Fig — Each color indicates a highly correlated sub-flock inside the overall flock using the k-means method. (PDF) [file pcbi.1010869.s004.pdf]

Sub-groups induced by fluctuation vectors

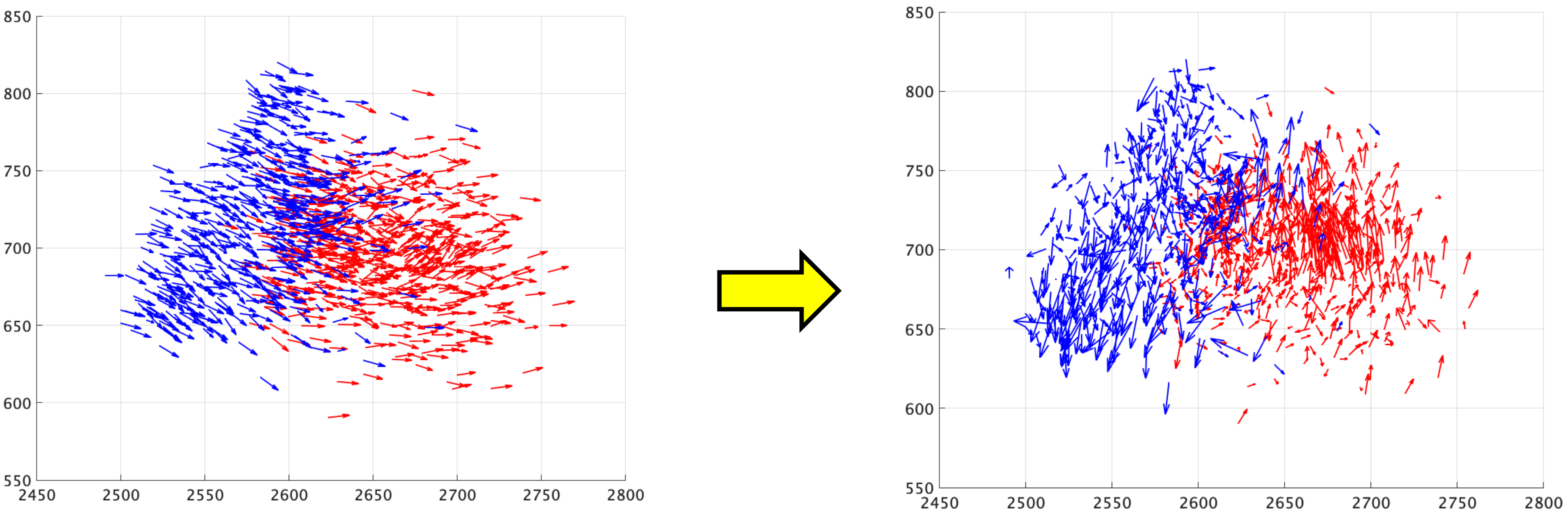

Velocity vectors projected onto x-y plane

Fluctuation vectors projected onto x-y plane
