# Supplementary material for: Functional duality in group criticality via ambiguous interactions
Source: PLoS Comput Biol. 2023 Feb 15;19(2):e1010869. doi: 10.1371/journal.pcbi.1010869 (PMC9931117; doi:10.1371/journal.pcbi.1010869)
Supplement: S6 Fig — (PDF) [file pcbi.1010869.s006.pdf]

Partial information decomposition (PID)

$$\underbrace{I(\{\boldsymbol{X}_1, \boldsymbol{X}_2\}, K)}_{\text{Mutual information}} = \underbrace{R(\boldsymbol{X}_1, \boldsymbol{X}_2; K)}_{\text{Redundancy}} + \underbrace{U_1(\boldsymbol{X}_1, \boldsymbol{X}_2; K)}_{\substack{\text{Unidirectional flow} \\ \text{Group 1 to K}}} + \underbrace{U_2(\boldsymbol{X}_1, \boldsymbol{X}_2; K)}_{\substack{\text{Unidirectional flow} \\ \text{Group 2 to K}}} + \underbrace{S(\boldsymbol{X}_1, \boldsymbol{X}_2; K)}_{\text{Synergy}}$$

where $\boldsymbol{X}$ is $\boldsymbol{V}$ or $\boldsymbol{F}$

Redundancy

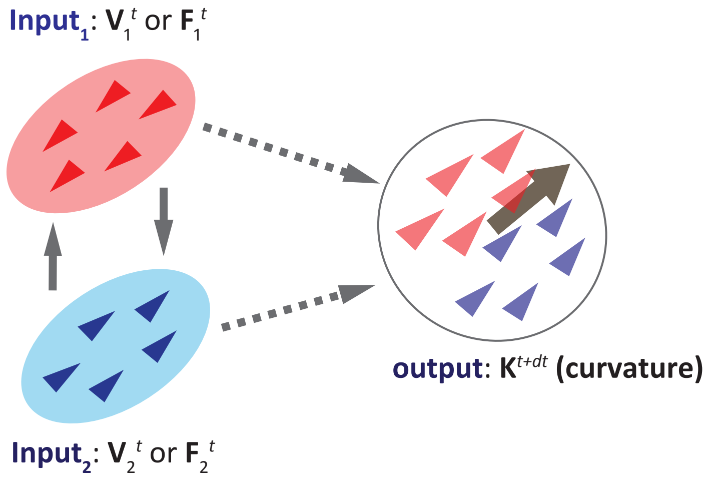

Either of the inputs may effect on the result

Unique 1

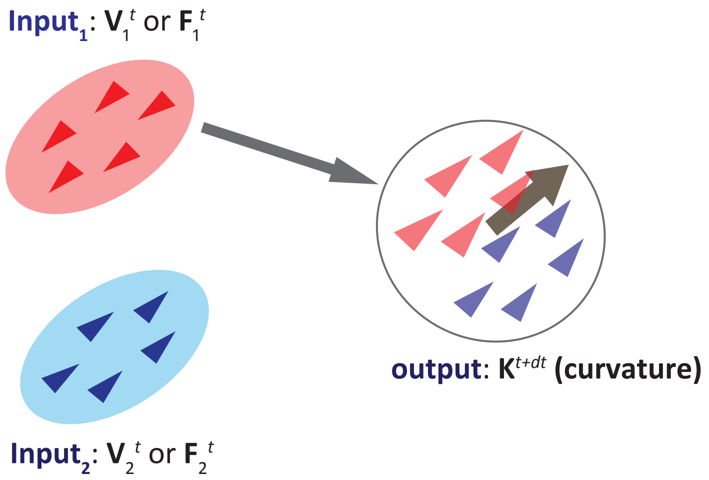

The group 1 only effects on the result

Unique 2

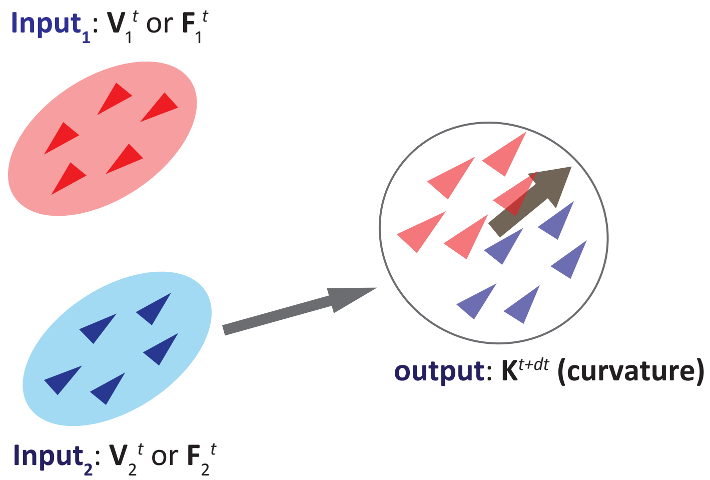

The group 2 only effects on the result

Synergy

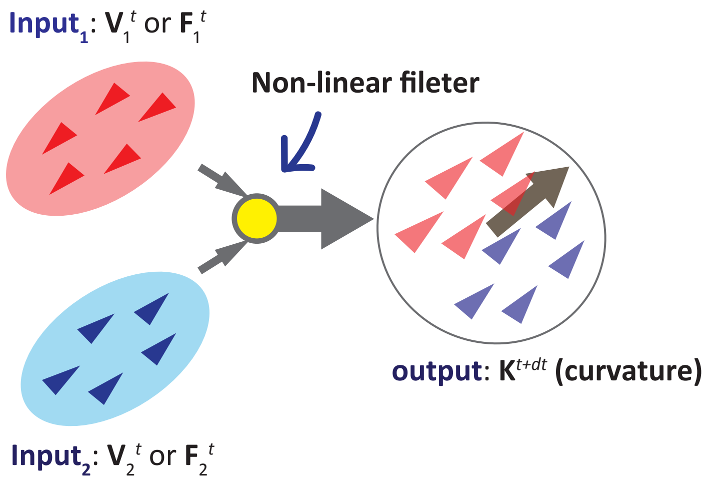

Both the inputs effect on the result
