# Supplementary material for: Functional duality in group criticality via ambiguous interactions
Source: PLoS Comput Biol. 2023 Feb 15;19(2):e1010869. doi: 10.1371/journal.pcbi.1010869 (PMC9931117; doi:10.1371/journal.pcbi.1010869)
Supplement: S1 Appendix — (PDF) [file pcbi.1010869.s008.pdf]

Supporting Information

January 28, 2023

1 α-shape

The border individuals of the given flock must be defined to estimate the flock's volume. Because the flock is typically non-convex, the convex-full method is not adequate to determine the flock shape.

In this study, we apply the 'α-shape algorithm' [1] based on [2]. α is a parameter for detecting Delaunay triangulation complexes. The complexes become disconnected if α is too small, and if α is too large, the precision of non-convex is lost. In this study, we changed the α values because the shape of the flock was not stable. We set the average distance with the nearest neighbors $l(t)$ as alpha at time t.

2 Cover function : $\mathrm{Cover}(\boldsymbol{p}_1, \boldsymbol{p}_2, ..., \boldsymbol{p}_n)$

The cover function returns the minimum cap C on SP, which covers given points $\boldsymbol{p}_1, \boldsymbol{p}_2, ..., \boldsymbol{p}_n \in \mathsf{SP}$. This cap C is uniquely determined by the solid angle Θ along with the center vector $\boldsymbol{c}(\Theta)$. Mathematically,

$$\mathsf{C} = \{\boldsymbol{r} \in \mathsf{Sp} | \mathrm{dist}_{\mathsf{Sp}}(\boldsymbol{c}(\Theta), \boldsymbol{r}) < \Theta\} \tag{1}$$

where $\mathrm{dist}_{\mathsf{Sp}}(\boldsymbol{a}, \boldsymbol{b})$ is the minimum geodesic distance on the sphere Sp. Θ is the minimum solid angle along vector $\boldsymbol{c}(\Theta)$ which covers all $\boldsymbol{p}_1, \boldsymbol{p}_2, ..., \boldsymbol{p}_n$. Mathematically, $\Theta = \inf_k\{\theta_k \in (0, \pi) | \exists \boldsymbol{c}_k \in \mathsf{Sp}, \forall i \in \{1, 2, ..., n\}, \mathrm{dist}_{\mathsf{Sp}}(\boldsymbol{c}_k, \boldsymbol{p}_i) < \theta_k\}$. We define this function : $\mathsf{C} = \mathrm{Cover}(\boldsymbol{p}_1, \boldsymbol{p}_2, ..., \boldsymbol{p}_n)$

3 Details regarding the quasi-alignment algorithm

Rotation matrix : R

Let $\boldsymbol{v}, \boldsymbol{w}$ be the unit vector in $\mathbb{R}^3$. We have a cross product $\boldsymbol{x} = \boldsymbol{v} \times \boldsymbol{w}$ and an inner product $c = \boldsymbol{v} \cdot \boldsymbol{w}$. Then, the rotation matrix R is given as follows:

$$\mathrm{R} = \mathrm{E} + [\boldsymbol{x}]_\times + \frac{1}{1+c}[\boldsymbol{x}]_\times^2 \tag{2}$$

where $[\boldsymbol{x}]_\times$ is the skew-symmetric cross-product matrix of $\boldsymbol{x}$, that is, $[\boldsymbol{x}]_\times \boldsymbol{y} = \boldsymbol{x} \times \boldsymbol{y}$ for any $\boldsymbol{y}$.

Application of rotation matrix

Let the location of the agent i be $\boldsymbol{r}_i (\in \mathbb{R}^3)$ with its velocity $\boldsymbol{v}_i^t$ at time t. Let the past velocity vector be $\boldsymbol{v}_i^{t-\Delta t}$, where Δt is the time step, $\boldsymbol{v}_i^t = \boldsymbol{r}_i^t - \boldsymbol{r}_i^{t-\Delta t}$ and $\boldsymbol{v}_i^{t-\Delta t} = \boldsymbol{r}_i^{t-\Delta t} - \boldsymbol{r}_i^{t-2\Delta t}$. Then, we have a rotation matrix $\mathrm{R}_i^{\Delta t}$ from $\hat{\boldsymbol{v}}_i^t$ and $\hat{\boldsymbol{v}}_i^{t-\Delta t}$. The notation $\hat{\boldsymbol{x}}$ is the unite vector of $\boldsymbol{x}$. Therefore, the next expected agent i's direction is $\mathrm{R}_i^{\Delta t} \hat{\boldsymbol{v}}_i^t$. The predicted position is the current position added to the predicted position with its velocity, $\boldsymbol{r}_{\mathrm{pre},i}^{t+\Delta t} = \boldsymbol{r}_i^t + (V_{\max} \Delta t) \mathrm{R}_i^{\Delta t} \hat{\boldsymbol{v}}_i^t$.

Alignment prediction: $\boldsymbol{s}_j^t$

A set of predicted agent positions including the agent of interest itself, $\mathsf{X}^{\Delta t}$, is defined as $\{\boldsymbol{r}_{\mathrm{pre},j}^{t+\Delta t} | j \in \mathsf{N}_i \cup \{i\}\}$ for each layer Δt. Then, we find the minimum Δt whose all predicted positions exceed SP_i, that is, $|\boldsymbol{r}_{\mathrm{pre},j}^{t+\Delta t} - \boldsymbol{r}_i^t| > R_{max}^i$ for all $j \in \mathsf{N}_i \cup \{i\}$. We compute Δt up to 20 steps before the current step.

The prediction points $\boldsymbol{s}_j^t$ on the interaction sphere are provided by applying $\mathsf{X}^{\Delta t_{\min}}$. These points $\boldsymbol{s}_j^t$ on the interaction sphere represent the intersection on SP_i from $\boldsymbol{r}_i^t$ to each point in $\mathsf{X}^{\Delta t_{\min}}$ (see Fig A).

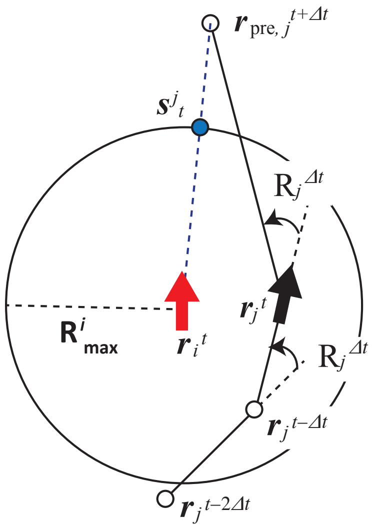

Figure A: **Alignment prediction using the rotation matrix.** i (red) is the agent of interest. j ($\in \mathsf{N}_i \cup \{i\}$) is its neighbour. The predicted point $\boldsymbol{s}_j^t$ on the sphere SP_i is the vector intersection, $\boldsymbol{r}_{\text{pre},j}^{t+\Delta t} - \boldsymbol{r}_i^t$, when Δt is the minimum time that satisfies $|\boldsymbol{r}_{\text{pre},j}^{t+\Delta t} - \boldsymbol{r}_i^t| > R_{max}^i$ for all $j \in \mathsf{N}_i \cup \{i\}$.

4 Noise function : von Mises–Fisher distribution

von Mises–Fisher distribution

The von Mises–Fisher distribution is the Gaussian distribution on the sphere [3, 4]. Because the sphere has a periodic boundary, the Gaussian distribution cannot be applied to this plane. In this study, we used random directions derived from the given sphere cap C. To ensure computational efficiency, the central axis $\boldsymbol{c}(\varphi, \theta)$ of C shifts to the $z-$axis. We only apply von Mises–Fisher distribution to the polar angle θ for the sake of simplicity. For the azimuthal angle φ, we apply a uniform distribution. Therefore, we only have to consider the von Mises distribution in directional statistics [5].

The von Mises probability density function for the angle θ is given as

follows:

$$f(\theta|\mu,\kappa) = \frac{e^{\kappa\cos(\theta-\mu)}}{2\pi I_0(\kappa)} \tag{3}$$

where $I_0(\kappa)$ is an order 0 modified Bessel function. μ is the mean value (in our model, the cap center $\boldsymbol{c}(\Theta)$), and $1/\kappa$ is the variance (in our model, the polar angle Θ) in the Gaussian context.

Noise function : $\mathrm{Rand}_{\mathrm{VM}}(\Theta, \boldsymbol{c}(\Theta))$

To determine the random point on the sphere SP, we require two outputs: azimuthal angle ϕ and polar angle θ. To compute the former (i.e., the azimuthal angle), we simply calculated the random value within a certain interval, $[0, 2\pi]$. To calculate the latter (i.e., the polar angle), we used the von Mises distribution. We assumed the mean value $\mu = 0$ and κ to be Θ (i.e. random distribution around $(0, 0, 1)$-axis at this stage). As a random function, we used Python's `np.random.vonmises`. Since the solid angle Θ cannot directly apply κ, we defined the empirical function $\kappa = f(\Theta)$ to correspond to an appropriate value. After the random point was determined, say (ϕ, θ), the vector space was rotated along $\boldsymbol{c}(\Theta)$ to obtain the aimed result.

We named this random function $\mathrm{Rand}_{\mathrm{VM}}$. The algorithm of $\mathrm{Rand}_{\mathrm{VM}}$ is listed in Fig B.

Algorithm : $(\phi, \theta) = \mathrm{Rand}_{\mathrm{VM}}(\Theta, \boldsymbol{c}(\Theta))$

Data: $\Theta, \mathbf{c}(\Theta)$

; /* Θ:solid angle, $\mathbf{c}(\Theta)$:the center vector in C */

Result: (ϕ, θ)

$\kappa \xleftarrow{f} \Theta$

$\phi' = \mathrm{random.uniform}(0, 2\pi)$

$\theta' = \mathrm{random.vonmises}(0, \kappa)$

; /* Rotate along $\mathbf{c}(\Theta)$ */

$(\phi, \theta) \leftarrow (\phi', \theta')$

Figure B: **Random distribution algorithm:** $\mathrm{Rand}_{\mathrm{VM}}$.

5 Mutual diffusion

In addition to supper diffusion, mutual diffusion is used for evaluating internal behavior. Mutual diffusion indicates the amount by which individuals in the group move with respect to one another.

$$\delta r_m^2(t) = \frac{1}{T-t}\frac{1}{N}\sum_{t_0=0}^{T-t-1}\sum_{i=1}^{N}[|\boldsymbol{s}_{ij}(t+t_0)| - |\boldsymbol{s}_{ij}(t)|]^2 \tag{4}$$

where $\boldsymbol{s}_{ij} = \boldsymbol{r}_j - \boldsymbol{r}_i$, and j is the nearest neighbor of i at time t_0. $\delta r_m^2(t)$ is also proportional to t^{α_m}. The parameter slope α_m must be below those of supper diffusion α because the group formation must have a stable formation regardless of neighbor shuffling. This implication is valid for our model. The effect of supper diffusion suppresses that of mutual diffusion.

6 Neighbour shuffling and border diffusion

A high internal fluctuation is also observed in Fig Ca. The graph shows the shuffling neighbor rate with time, $Q_M(t)$, where $M_i(t)$ is the number of neighbors of agent i at time $t + t_0$ that share the same neighborhood. $Q_M(t)$ is given as follows:

$$Q_M(t) = \frac{1}{N}\sum_{i \in \mathsf{N}} \frac{M_i(t)}{M} \tag{5}$$

We found that the decay of $Q_M(t)$ is significantly faster than the experimental one [6]: the high diffusive process was also confirmed. The effective dimension $\hat{d} \approx 2.5$ (approximated by $M \sim aR^{\hat{d}}$, where R is the radius) is higher than of the real flocks ($\hat{d} \approx 2.3$ [6]). In addition, as noted above, higher values are noted in the case of mutual diffusion as compared to the case of the real flock.

This difference mainly occurs because the real flock has an additional constraint on the gravitational axis: the real flock shows a weaker diffusion in the vertical direction than in the horizontal direction. This asymmetrical relation suppresses the overall diffusion. In contrast, our model has no such constraints. The flock's motion does not depend on any particular axis and the effective dimension has a high value as a result.

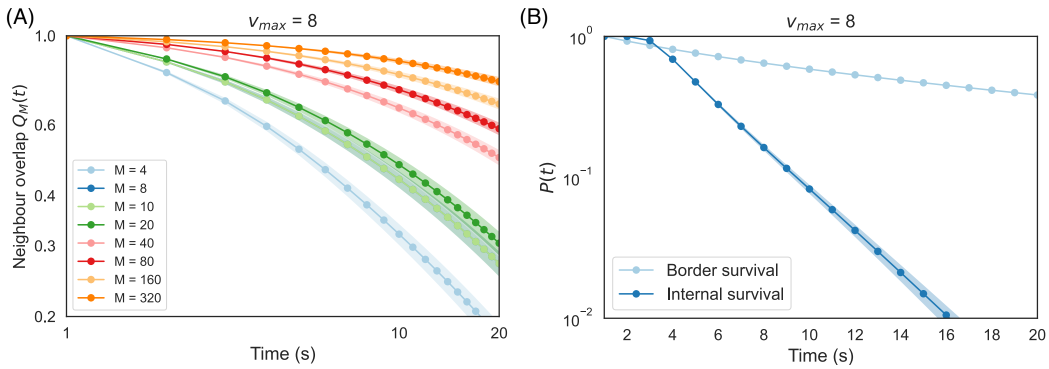

Figure C: **Neighbor shuffling and border diffusion.** (a) Neighbor shuffling rate for each neighbor set. (b) The internal and border survival probability. Internal diffusion is significantly faster than border diffusion. Each characteristic time ($\lambda_{\text{border}} \approx 21.9$ and $\lambda_{\text{internal}} \approx 3.8$) can be computed via exponential approximation, $e^{-t/\lambda}$. Both are approximated using an exponential function. The internal diffusion decays faster than the exponential diffusion, whereas boundary diffusion decays slower than the exponential diffusion (see other parameter settings in S2 Fig.

Despite these high diffusion properties, the internal diffusion and border diffusion processes are essentially different. Cavagna et al.[2] reported that the border diffusion of starlings is significantly slower than the internal diffusion. Border diffusion is described as the survival probability of the member on the borders of α-shape at time t. In contrast, internal diffusion is described as the survival probability of the moving distance over average distance l_B (distance between the border and first internal nearest neighbors).

Fig Cb shows that the border diffusion is slower than the internal diffusion in our model (six times slower for the case of exponential approximation). The agent in our model remains at the edge of the flock for a longer duration than that inside of the flock. Note that this asymmetrical relation is not due to physical or ecological requirements but is a result of their interaction. Interestingly, despite the high neighbor shuffling compared with the real flocks, the long border survival tendency remains the same as that observed in nature.

7 Correlation function on speed fluctuation

The correlation function on speed fluctuation $C_{sp}(r)$ is given as follows:

$$C_{sp}(r) = \frac{1}{c_0} \frac{\sum_{i,j \in \mathsf{N}} \varphi_i \varphi_j \delta(r - r_{ij})}{\sum_{i,j \in \mathsf{N}} \delta(r - r_{ij})} \tag{6}$$

where φ_i is the scalar velocity fluctuation: $\varphi_i = |\boldsymbol{v}_i| - \sum_{j \in \mathsf{N}} |\boldsymbol{v}_j|$ and c_0 is a normalization parameter. Therefore, the correlation length of speed ξ_{sp} is satisfied where $C_{sp}(r = \xi_{sp}) = 0$.

8 Parameter settings

Below is a list of notations we used in our algorithm.

References

[1] Herbert Edelsbrunner and Ernst P. Mücke. Three-dimensional alpha shapes. *ACM Trans. Graph.*, 13(1):43–72, jan 1994.

[2] A. Cavagna, S. M. Duarte Queirós, I. Giardina, F. Stefanini, and M. Viale. Diffusion of individual birds in starling flocks. *Proceedings of the Royal Society B: Biological Sciences*, 280(1756):20122484, 2013.

[3] Philipp Berens. Circstat: A matlab toolbox for circular statistics. *Journal of Statistical Software, Articles*, 31(10):1–21, 2009.

[4] G. Kurz, Igor Gilitschenski, F. Pfaff, Lukas Drude, U. Hanebeck, Reinhold Häb-Umbach, and R. Siegwart. Directional statistics and filtering using libdirectional. *Journal of Statistical Software*, 89(4):1–31, 2019.

[5] N. I. Fisher. *Statistical Analysis of Circular Data.* Cambridge University Press, 1993.

[6] Andrea Cavagna, Lorenzo Del Castello, Irene Giardina, Tomas Grigera, Asja Jelic, Stefania Melillo, Thierry Mora, Leonardo Parisi, Edmondo Silvestri, Massimiliano Viale, and Aleksandra M. Walczak. Flocking and turning: a new model for self-organized collective motion. *Journal of Statistical Physics*, 158(3):601–627, Feb 2015.

Table A: Summary of the notation used in this paper.

Symbol	Meaning
**Sets**	
N	a set of all agents
N_i	a set of agent i's Delaunay neighbours
SP_i	an agent i's interaction sphere
$\mathsf{C}_{\mathsf{atr},i}$	an agent i's sphere cap induced by quesi-attaraction
$\mathsf{C}_{\mathsf{alg},i}$	an agent i's sphere cap induced by quesi-alignment
$\mathsf{C}_{\mathsf{avd},i}$	an agent i's sphere cap induced by avoidance
$\partial\mathsf{C}_{\mathsf{avd},i}$	the border of $\mathsf{C}_{\mathsf{avd},i}$
C_i	an agent i's interaction sphere generated by $\mathsf{C}_{\mathsf{atr},i}$ and $\mathsf{C}_{\mathsf{alg},i}$
**Scalars**	
N	the number of agents (i.e. $\lvert\mathsf{N}\rvert$)
$V_{\max}$	max velocity
R	repulsion radius
$R^i_{\max}$	a radius of SP_i
K	a mean curvature
**Vectors**	
$\boldsymbol{r}_i$	an agent i's position vector
$\boldsymbol{c}_i$	an agent i's center of mass on $\mathsf{C}_i^{\mathrm{atr}}$ or $\mathsf{C}_i^{\mathrm{alg}}$
$\boldsymbol{t}_j$	a target vector on SP_i ($\forall i \in \mathsf{N}_i$)
$\boldsymbol{s}_j$	a predicted vector outside SP_i ($\forall i \in \mathsf{N}_i$)
$\boldsymbol{v}_i$	an agent i's velocity vector
$\boldsymbol{u}_i$	an agent i's fluctuation vector
**Matrixes**	
$\mathrm{R}_i^{\Delta t}$	an agent i's rotation matrix at Δt steps
**Functions**	
$\mathrm{dist}(\boldsymbol{x}, \boldsymbol{y})$	the Euclidean distance between $\boldsymbol{x}$ and $\boldsymbol{y}$
$\mathrm{dist}_{\mathsf{SP}}(\boldsymbol{x}, \boldsymbol{y})$	the minimum distance between $\boldsymbol{x}$ and $\boldsymbol{y}$ on SP
$\mathrm{Cover}(\boldsymbol{p}_1, \boldsymbol{p}_2, ..., \boldsymbol{p}_n)$	the minimum cone which covers all $\boldsymbol{p}_1, \boldsymbol{p}_2, ..., \boldsymbol{p}_n (\in \mathsf{SP})$.
$\mathrm{Rand}_{\mathrm{VM}}(\Theta, \boldsymbol{c}(\Theta))$	generate the random point from C in SP
