# Supplementary material for: Functional duality in group criticality via ambiguous interactions
Source: PLoS Comput Biol. 2023 Feb 15;19(2):e1010869. doi: 10.1371/journal.pcbi.1010869 (PMC9931117; doi:10.1371/journal.pcbi.1010869)
Supplement: Attachment — Submitted filename: Reviewer_plos__1_.pdf [file pcbi.1010869.s012.pdf]

Reviewers' Comments and Authors' Responses

"**Functional duality in group criticality via ambiguous interactions**"

We thank the reviewers and the editor for their feedback on our original manuscript. We have fully revised the manuscript according to the reviewer recommendations, and we hope that these changes and additions have addressed all concerns.

Reviewer #2

Reviewer #2: THis second version is, without a doubt, an improvement with respect the first version. But annoying mistakes remain in the use of the english language. For example in the first sentence of the DISCUSSION sentence .. *************************** In this study, we showed the nested criticality that emerged from the same algorithm, namely, the ambiguous interaction model, which is a natural extension of the representative flocking model. We re-interpreted the attraction and alignment as short- and long-term predictions, respectively, and as a key concept of our model, we considered these predictions as regions C rather than points. ************************************* The expression "same algorithm" makes the sentence so difficult to understand. also the expression "region C rather than points" ... could be written as "regions rather than points". The authors need a conceptual editor ....not a mere "native speaker". They need someone with experience in technical english ....***

- We apologize for our English manuscript still have grammatical and other issues. We asked them to once again check our English by native and editor experienced writer. We hope our revised text becomes more readable.

Reviewer #3

The authors replied reasonably to all arguments and defend their thesis soundly, it deserves publication as its. Thanks for providing the numerics in a type of source code and updating the text in almost all the issues raised before.

- Thank you for reviewing our paper. We are confident that the reviewer's remarks have strengthened the content of our paper.
