# Supplementary material for: Functional duality in group criticality via ambiguous interactions
Source: PLoS Comput Biol. 2023 Feb 15;19(2):e1010869. doi: 10.1371/journal.pcbi.1010869 (PMC9931117; doi:10.1371/journal.pcbi.1010869)
Supplement: Attachment — Submitted filename: Reviewer_plos__1_.docx [file pcbi.1010869.s011.docx]

Reviewers’ Comments and Authors’ Responses

“**Functional duality in group criticality via ambiguous interactions**”

We thank the reviewers and the editor for their feedback on our original manuscript. We have fully revised the manuscript according to the reviewer recommendations, and we hope that these changes and additions have addressed all concerns.

The main revisions are as follows:

1. Introduction: We have focused on two important topics: the nested criticality and its functional role. Nested criticality means that the system criticality is not a single-scale phenomenon but, rather, occurs across several scales. Most existing models focus on a single scale only when considering the criticality. The functional role of criticality is also an open question. In particular, previous explanations of the origin of group turns have relied on mechanisms other than the criticality (e.g., the quantum effect or a few individual fluctuations, although some researchers have noted a relationship between the two). Our study aims to consider nested criticality and its functional role using our proposed model, which is a natural extension of previous models such as the Boids model.
2. Material and Methods: We have added two figures (Figs. 1A and 1B) to aid reader understanding of our algorithms. We have replaced overly complex mathematical explanations with intuitive descriptions and moved the exact definitions to the Supporting Information (S1 Appendix; we have also added one new figure and one new pseudo-code for our algorithm). Fig. 2 has also been revised.
3. Results: We have changed the overall structure of this section. First, to clarify the connection between the arguments, we now discuss micro-level criticality (Lévy walk and 1/*f* fluctuation). The discussions of border diffusion and neighbor shuffling, which are not directly related to micro-level criticality, have been moved to S1 Appendix. We have added a further explanation of *k*-means clustering with a new pseudo-code figure to the “Information transfer between correlated groups” section (Fig. 8). Furthermore, we have entirely revised the explanation of the continuous PID and the related results.
4. Discussion: We have fully revised the Discussion section, adding new references, which were suggested by the reviewers, and associated discussion. We have rewritten unclear text identified by the reviewers and related it to the issues discussed in the Introduction. In addition, the difference between our proposed model and existing models have been presented, along with the limitations of our study.
5. Supporting Information: We have included the Python codes of our algorithm (with explanations of each function) to enable readers to run and check our results. By running *model.py* with an appropriate environment, users can obtain flock movies and data. Users can also adjust the parameters beyond those considered in our study (e.g., to set a max turning rate for a more realistic movement).

Reviewer #1

***They do not attempt to re-create exact animal behaviors, but rather a simple model that can reproduce the observed phenomena. I recomend to reject this article, as I find the writing hard to follow, and the detail of the methods applied lacking. There is a general lack of articles (an, a the) throughout the paper. Spelling mistakes such as supper instead of super.***

- We apologize for the difficulty experienced in reading our original paper. The revised manuscript has been professionally edited by a native speaker, and thus, we hope that it is now highly readable. We have also fully restructured the paper and entirely rewritten the Introduction, Materials and Methods, and Discussion sections. Furthermore, we added the codes of our algorithm to the Supporting Information (S2 Appendix). The algorithms, which were of concern to the reviewer, have been added to the main text and S1 Appendix.

***It difficult to understand parts of the manuscript as lines 130-134, line 144, line 200 (the von distribution ??)***

- We apologize for the complex explanation of our algorithm provided in the original paper. The revised paper contains two new figures (Figs. 1A and 1B), as does the S1 Appendix. We have also reduced the number of complex mathematical expressions so that the key points can be understood from the figures (the exact definitions have been moved to information in S1 Appendix, to aid reader understanding).

***For the methods, some quantities, e.g. mu only appears on line 201, then not until the caption of Figure 7. Similarly for kappa, which only appears on line 201, without describing what it is. Combinging this writing with the very compact mathematical notation for the model, such as equation 2 and 3 along with the long notation in the explanation of these equations makes the paper difficult to understand.***

- We apologize that our original paper contained many incorrect descriptions and inappropriate symbol usage. For instance, the parameters *μ* and *κ* were intended to be applied in the von Mises‒Fisher distribution, whereas the other *μ* term indicated the slope of the Lévy walk. In the revised paper, we explain the von Mises‒Fisher distribution in S1 Appendix and define the random function Random(Θ, ***c***(Θ)) to avoid this confusion. The algorithm of this function is included in S1 Appendix.

***It also does not appear from the paper how a Partial Information decomposition is done. In a similar vein it would be nice with just a brief mention of how the k-means partitioning of the flock into two subgroups was preformed, to make this a stand-alone piece of research.***

- Again, we apologize for the lack of clarity in the original paper. The subsection “Information transfer between correlated groups” has been entirely revised. In particular, we have added an explanation regarding the k-means method using a pseudo-code algorithm (as we implement the k-means method using a Matlab function, we only refer to its source). Furthermore, we have added a new explanation to the “Partial information decomposition on a rapid group turn” subsection. To implement the PID, we also used the Matlab code given in <https://github.com/robince/partial-info-decomp>.

***There is new information presented in the discussion, such as line 551: ''The agents of our model have intact visual fields,''. The conclusion is also extremely short, and does not provide much information on the exact value of this model over other models.***

- As mentioned above, the Discussion section has been completely rewritten. Thus, the sentence noted by the reviewer has been eliminated. While revising our paper, we reviewed the author guidelines and noted that *PLOS Computational Biology* does not have a “Conclusion” section. Therefore, we have revised the Discussion section.
- The exact value and the limitation of our model have also been added to the final paragraph of the Discussion: “In this study, we considered only one parameter, v_max_/R. It remains to be seen whether the same results can be obtained by adding more realistic constraints (e.g., a maximum turning rate, visual field, and gravity effect) to our model. However, the behavior of our model is statistically close to those of real flocks. We also note that the realistic conditions implemented using the Boids model cannot replicate a more accurate description of the group criticality [41]. Furthermore, few models focus on criticality across several levels. The effect of adding furthermore realistic assumptions to our model leaves to future study.”

***It would have been nice with a clearer statement on which aspects this model captures better, or if it fits more accurately to observational data (I know the authors explicitly says they do not compare with real data, but this would lift the impact of this manuscript.), of it this new model captures mode different critical behaviors than other models of flocking.***

- This study certainly does not completely reproduce an actual flock's movements; however, this is a matter of degree. Most flock models do not even reproduce the various properties reported in our study (Ros et al., 2011; Morrin et al., 2015; Hemelrijik et al., 2015; Gerlee et al., 2017; Sonoda et al., 2019; Strombom et al., 2021). For example, Hemelrijik et al. (2015) introduced even more "realistic" interactions to their model for scale-free correlation than that of our study, as we have mentioned above. However, our results more closely agree with the experimental results. Furthermore, none of the existing models have successfully reproduced Lévy walks in a flock. In this context, our model more realistically reproduces herd movements than other models.

Reviewer #2

***This manuscript examines a clever extension of the theory of flock dynamics and finds that many collective behaviors can be deduced from the extension proposed. The manuscript presents, a somewhat complicated version, of the alignment and attraction model used. The main point of the manuscript can be found (at last!) in the following line that explains an interesting point: What is alignment? The authors present an interesting extension of this concept that has some overtones with the concept of Anticipation. Or in line 132“In these contexts, our claim is clear: both interactions stem from a common interaction. ““We showed that he interaction between alignment and attraction, having been applied to many flocking models, can be regarded as a single interaction in terms of time-scale differences. The alignment indicates the infinity long predicted position of the neighbors, whereas attraction indicates the interaction with the current position of the neighbors. We showed that the medium region of this time scale could play a critical role in group criticality. However, our result is restricted to the two-dimensional condition. The conclution are interesting …and could be generalized to other situations different from flock formation. The papers suffers from some (resolvable) problems in the exposition.***

- We apologize for your difficulty in reading our original manuscript. The revised manuscript has been professionally edited by a native speaker and, thus, we hope that it is highly readable. We have fully restructured the paper and fully rewritten the Introduction, Materials and Methods, and Discussion sections. Furthermore, we have added the codes of our model to the Supporting Information (S2 Appendix). The algorithms, which were of concern to the reviewer, have been added to the main text and S1 Appendix.

***a very vert minor point …Dr. Niizato does not have an institutional email? (***[***t_niizato@yahoo.co.jp***](mailto:t_niizato@yahoo.co.jp)***) this is strange***

- We have replaced Dr. Niizato’s personal e-mail address with his institutional e-mail address.

***the exposition of the problem, the main arguments, even the equations is complex and difficult to follow. An particularly nasty example is equation 5… where they explain the symbol xi(t) ..that does not appear directly into the equation!***

- We apologize that our original manuscript contained many incorrect descriptions and inappropriate symbol usage. The Materials and Methods section has been completely written. We have also added two figures (Figs. 1A and 1B), which provide an intuitive overview of our algorithm. The details of the complex mathematical expressions have been moved to S1 Appendix (we have also added one figure and one pseudo-code to this appendix); thus, this section in the main manuscript can now be understood without considering the exact mathematical expressions.

***3)*** ***The overall exposition is very non-direct. The first page of the introduction does not give any clue of what the authors are planning to do with the Flock problem. In the discussion section the first paragraph is misleading (In this study, we discussed various critical phenomena of collective behavior produced by ambiguous interactions by constructing a model of ambiguous interactions based on the natural extension of the representative flocking model: quasi-attraction and quasi-alignment defined as short- and long-term predictions, respectively.” .. In fact they discuss one/two parametrs of a very restricted model of a very restricted problem. Also in the Conclusion (The flock has dual aspects: a force distribution as fluctuation and a force converter as the group morphology (i.e., heterogeneous velocity distribution)….. the redaction is awkard it should say “Flock MODELS have two aspects ….”)***

- The exact value and the limitation of our model are now discussed in the final paragraph of the manuscript: “In this study, we considered only one parameter, v_max_/R. It remains to be seen whether the same results can be obtained by adding more realistic constraints (e.g., a maximum turning rate, visual field, and gravity effect) to our model. However, the behavior of our model is statistically close to those of real flocks. We also note that the realistic conditions implemented using the Boids model cannot replicate a more accurate description of the group criticality [41]. Furthermore, few models focus on criticality across several levels. The effect of adding furthermore realistic assumptions to our model leaves to future study.” In line with this change, other general statements have been weakened throughout the Discussion.

***In general the text will benefit from a CONCEPTUAL editor. The authors do themselves weak favor with their text. For example this sentence “Although the OR and AND functions guide the understanding on PID” people could differ. PID is vastly different from simple logic gates.***

- As the reviewer has noted, the explanation of the PID AND- and OR-gates is likely inappropriate. In the revised manuscript, we explain the continuous version of the PID in terms of the input correlation. In other words, the redundant system has a high input correlation for the same output, as we can restore the other input if one input is lost. In this sense, the redundant system contains the symmetric input. In contrast, the synergy has an asymmetric input, which means a non-linear effect on the output. The revised manuscript has been rewritten to contain all these intuitive but mathematically valid explanations (text in the “Partial information decomposition on a rapid group turn” subsection and the 5^th^ paragraph of the Discussion section).

Reviewer #3

***The authors contribute important work and insights in the area of collective animal (and in general 'agent') behaviour. They offer an extension of the standard 'boid' algorithm that addresses criticality as one of the most important but usually overlooked dynamical regimes necessary for sustainable collective behaviour. Therefore, this very well thought and worked out, paper deserves publication.***

- We highly appreciate the reviewer’s feedback and we feel that the reviewer has a deep understanding of our study. In accordance with all the reviewer comments and suggestions, we have fully restructured our paper and have entirely rewritten the Introduction, Materials and Methods, and Discussion sections. Furthermore, we have added the codes of our algorithm to the Supporting Information (S2 Appendix). The algorithms have been included in the main text as well as in the S1 Appendix.

***On this issue of criticality, I would like to point out to the authors another, maybe not so well known, publication that has addressed a certain extension of the standard Vicsek model for swarming based on a logistic map type of updating. What has been observed at that publication is the fact that sustainable, stable, swarming appears when the logistic-map updating self-organizes on the Feigenbaum critical point. Here is the reference:A. García Cantú Ros, Ch.G. Antonopoulos, V. Basios,Emergence of coherent motion in aggregates of motile coupled maps,Chaos, Solitons & Fractals, Volume 44, Issue 8, 2011, Pages 574-586, ISSN 0960-0779,***[***https://doi.org/10.1016/j.chaos.2011.05.005***](https://doi.org/10.1016/j.chaos.2011.05.005)***.(***[***https://www.sciencedirect.com/science/article/pii/S0960077911000683***](https://www.sciencedirect.com/science/article/pii/S0960077911000683)***). The above reference might help the authors find a relationship between critical phenomena and group dynamics related to their important quest and as they quote on <association between individual criticality (i.e. Lévy-walk) and group criticality (i.e. scale-free correlation), and how these group criticalities relate to collective turns. I think that it will add to their discussion if they consider a connection with the above-mentioned paper. But this is only out of collegial academic interest since, I would strongly suggest their work to be published even if they choose not to discuss briefly or even mention this reference.***

- Thank you for suggesting this important study, of which we were unaware. In the revised Introduction, we focus on two topics (i.e., nested criticality and its functional role), and we discuss Ros et al. study in the 3^rd^ paragraph of the Discussion section in the context of nested criticality. We argue that micro-level criticality, including weak chaos and inverse Bayesian inference, contribute to system robustness rather than to system stability. We regard the flock system to be robust because the flock does not have an original state but an ever-changing dynamic state that depends on its environment. This aspect is important for micro-level weak chaotic systems. As regards micro-level criticality, each agent is susceptible to external stimuli and its role within the group is unstable. We have added a relevant statement to the revised Discussion.

***Their study is also interestingly original as they bring forth detailed relations between scale-free correlations and collective rotations, this something new as far as I know. They also provide the pseudocode for their simulations, as the journal's policy requires. Personally, I would be happier if a free and open code was provided, lie an octave or R-code. It is up to the authors to add this.***

- In the revised manuscript, we have added two new pseudo-codes to the main text (for the k-means method) and S1 Appendix (the von Mises‒Fisher random function). Following the request of the reviewer, we have added the Python code of our model with explanations for each function (S2 Appendix). Readers can run and check our algorithm in an appreciate environment.

***Another connection would be to discuss the authors' important work and take on the implications that it has for inference as it is put forth here (Gunji Yukio-Pegio, Murakami Hisashi, Tomaru Takenori and Basios Vasileios, 2018, Inverse*** ***Bayesian inference in swarming behaviour of soldier crabs, Phil. Trans. R. Soc. A.3762017037020170370). But again this is not to be taken as a requirement for adding any new reference, but rather for future discussion and work. The paper is indeed opening new ground for such investigations, and therefore it merits publication even as is.***

- Thank you for suggesting this reference. As noted above, we have added this reference to the Discussion in the context of the nested criticality.
